# A promising high-energy-density material

**DOI:** 10.1038/s41467-017-00286-0

**Published:** 2017-08-03

**Authors:** Wenquan Zhang, Jiaheng Zhang, Mucong Deng, Xiujuan Qi, Fude Nie, Qinghua Zhang

**Affiliations:** 1 Research Center of Energetic Materials Genome Science, Institute of Chemical Materials, China Academy of Engineering Physics (CAEP), Mianyang, 621000 China; 2 School of Materials Science and Engineering, Shenzhen Graduate School, Harbin Institute of Technology, Shenzhen, 518055 China; 3 Sichuan Co-Innovation Center for New Energetic Materials, Southwest University of Science and Technology, Mianyang, 621010 China

## Abstract

High-energy density materials represent a significant class of advanced materials and have been the focus of energetic materials community. The main challenge in this field is to design and synthesize energetic compounds with a highest possible density and a maximum possible chemical stability. Here we show an energetic compound, [2,2′-bi(1,3,4-oxadiazole)]-5,5′-dinitramide, is synthesized through a two-step reaction from commercially available reagents. It exhibits a surprisingly high density (1.99 g cm^−3^ at 298 K), poor solubility in water and most organic solvents, decent thermal stability, a positive heat of formation and excellent detonation properties. The solid-state structural features of the synthesized compound are also investigated via X-ray diffraction and several theoretical techniques. The energetic and sensitivity properties of the explosive compound are similar to those of 2, 4, 6, 8, 10, 12-(hexanitrohexaaza)cyclododecane (CL-20), and the developed compound shows a great promise for potential applications as a high-energy density material.

## Abstract (web summary)

High energy density materials are of interest, but density is the limiting factor for many organic compounds. Here the authors show the formation of a high density energetic compound from a two-step reaction between commercially available compounds that exhibit good heat thermal stability and detonation properties.

## Introduction

Over the past decades, the discovery and development of compounds known as high-energy density materials (HEDMs) has been of continued interest to the chemical science community^1–10^. Density is one of the crucial properties of HEDMs because it greatly affects the detonation velocity of energetic materials and, even more importantly, the detonation pressure increases with the square of the density^9^. In this context, the development of HEDMs with even higher density is extremely desirable^9, 11^. However, it is established that there exists a density limit for organic molecules based on carbon (C), hydrogen (H), nitrogen (N), and oxygen (O), which makes the discovery of HEDMs with a density as high as 2.0 g cm^−3^ particularly challenging^9, 11, 12^. In addition, for most HEDMs, the energy density and the molecular stability have an inverse relationship, hence further increasing the difficulty in developing HEDMs with high density^13^.

In the quest for better detonation-performing HEDMs, a large number of CHNO-based high explosives have been developed; however, only in a few instances, there have been reports of crystal densities as high as 2.0 g cm^−3^ (refs ^9–11^). A well-known example is 2,4,6,8,10,12-(hexanitrohexaaza)cyclododecane (CL-20), a tremendously powerful explosive with a crystal density of 2.035 g cm^−3^ (only for ε-CL-20) at room temperature^14^. In particular, ε-CL-20 is extremely sensitive to mechanical stimuli (shock and friction); hence, it has be handled with enormous care. Furthermore, 1,3,4,6-Tetranitroglycouril (TNGU) and 1,3,4,5,7,8-hexanitrooctahydrodiimidazo[4,5-*b*:4′,5′-*e*]pyrazine-2,6(1*H*,3*H*)-dione (HHTDD) are two examples of super-high density HEDMs containing N-nitrourea functionality, and have the crystal densities of 2.03 and 2.07 g cm^−3^, respectively. However, high reactivity to water limits their applications^15^. Recently, another HEDM, [1,2,3,4]tetrazino[5,6-e]-[1,2,3,4]tetrazine1,3,6,8-tetraoxide (TTTO), has been reported, which has an estimated density of 1.98 g cm^−3^ and a detonation velocity of 9.71 km s^−1^ (refs ^16–19^). However, an extremely complex synthesis procedure and a low resistance to hydrolysis, limits its practical use. In other cases, heptanitrocubane (HpNC) and 2,4,6-tris(3′,5′-diamino-2′,4′,6′-trinitrophenylamino)-1,3,5-triazine (PL-1) have not only been found to be powerful HEDMs with a crystal density of 2.03 and 2.02 g cm^−3^, respectively but also suffering from similar problems of complicated synthesis^10, 20^. Although, the synthesis of these well-known HEDMs are significant breakthroughs in the field of energetic materials, the majority of them face similar problems, such as complex synthetic routes, high production costs and poor molecular stabilities (e.g., hydrolysis or high sensitivity). Therefore, a long-term challenge in this field is the design and synthesis of HEDMs that demonstrate the density as high as 2.0 g cm^−3^, low water solubility, a high detonation velocity comparable to CL-20, acceptable sensitivity toward accidental stimuli, and good thermal stability at higher than 200 °C. In addition, it is desirable that the HEDMs possess simple synthetic routes, low production costs, and easy scale-up^21, 22^.

Considering the above mentioned desired properties as guidelines for the rational design of new HEDMs, we present the facile synthesis of a promising high explosive, i.e., [2,2′-bi(1,3,4-oxadiazole)]-5,5′-dinitramide (named as ICM-101). The prepared compound is characterized by X-ray diffraction (XRD), infrared, multinuclear nuclear magnetic resonance (NMR) spectroscopy, elemental analysis and differential scanning calorimetry (DSC). In addition, quantum chemical calculations are used to predict the energetic properties of ICM-101.

## Results

### Synthesis of ICM-101

**Fig. 1:**
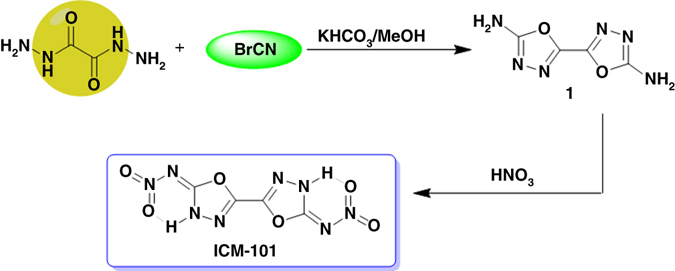
Synthesis route of ICM-101.

As shown in Fig. 1, ICM-101 can be readily obtained through two-step reactions from commercially available reagents. In the first step, an intermediate compound [2,2′-bi(1,3,4-oxadiazole)]-5,5′-diamine (**1**) was easily prepared from commercially available oxalyl dihydrazide and cyanogens bromide achieving a high yield of 90.5 wt%. Thereafter, a nitration reaction followed by treating **1** with fuming nitric acid for 24 h and then pouring the mixture into ice water. As a result, the target product ICM-101 precipitated from the solution as a white solid (yield: 66.8 wt%). This facile synthesis approach has proven to be successful, yielding a fresh example of CHNO-based HEDM with ultra-high density and adequate purity, and allows further scale-up to the production scale. It is encouraging that ICM-101 displays poor solubility in water, which is a highly desired property for practical formulation applications of insensitive munitions^23^.

### Crystal structure

**Fig. 2:**
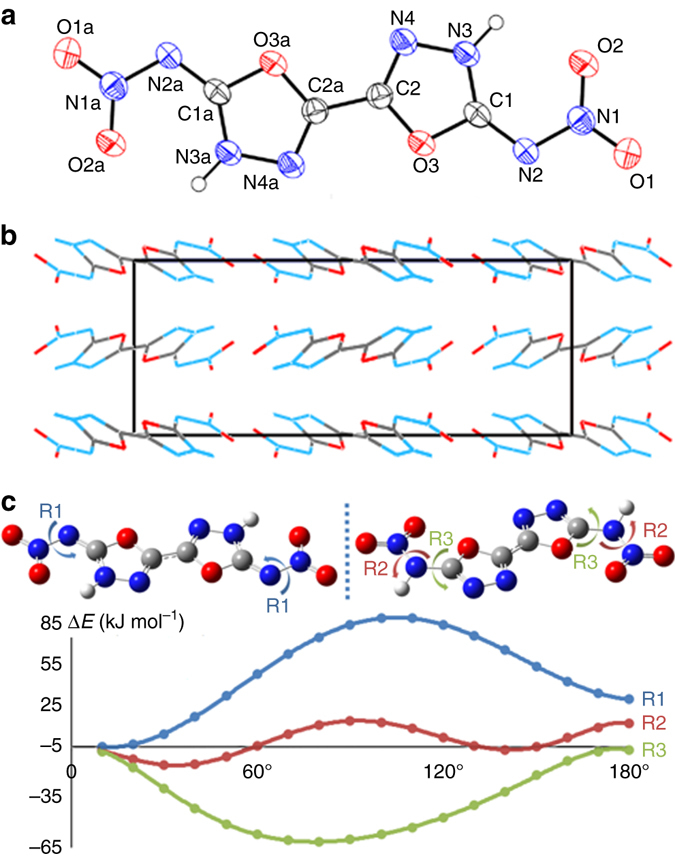
Crystal structure of ICM-101. **a** Single-crystal X-ray structure of ICM-101 with labeling (thermal ellipsoid plot: 30%). **b** Crystal packing of ICM-101 viewing down the unit cell axis **b**. **c** The relative energies of ICM-101 as a function of the rotation of the nitro groups (R1) in comparison with the relative energies of ICM-101 tautomer (protons bond to N2/N2a) as a function of the rotation of the nitro groups (R2) and nitroamine groups (R3). The initial structure for ICM-101 and its tautomer were obtained from crystal structure or set up as planar (rotation angle equal zero), respectively

Single crystals of ICM-101 suitable for XRD measurements were obtained by recrystallization from DMSO. The molecule crystallizes in the orthorhombic crystal system and belongs to the *Pbca* space group with four molecules per unit cell and an ultra-high crystal density of 2.037 g cm^−3^ at 170 K (a crystal density at 298 K is 1.99 g cm^−3^), and the detailed crystallographic data are provided in Supplementary Tables 1–4. Figure 2a shows the crystal structure. The bond lengths of C1-N2 (1.311 Å) and C1-N3 (1.331 Å) lie between average single and double C-N bonds (1.47 Å and 1.22 Å, respectively)^24^, which can be explained by a high degree of internal charge separation due to the big *π*-conjugated structure of the molecule^25^. As supported by the X-ray molecular structure, the protons are bonded to the nitrogen atoms (N3/N3a) in the oxadiazole ring and not located on N2/N2a. Such structural features result in intermolecular interactions between protons and O2/O2a, which fixes the rotation of nitro groups. Also, from the crystal structure, it can be found that the molecular structure is almost planar, while the dihedral angles between nitro groups and the bis(1,3,4-oxadiazole) plane are close to zero. More crystal packing information is provided in Supplementary Figs 1–3. In addition, the possible polymorph transitions of ICM-101 were also investigated using temperature-dependent XRD technology. Through the XRD patterns shown in Supplementary Figs 4 and 5, it can be found that there is no observable peak splitting or occurrence of new diffraction peaks over the temperature range of 30 to 180 °C, indicating that no polymorph transitions of ICM-101 occur throughout this heating process.

**Fig. 3:**
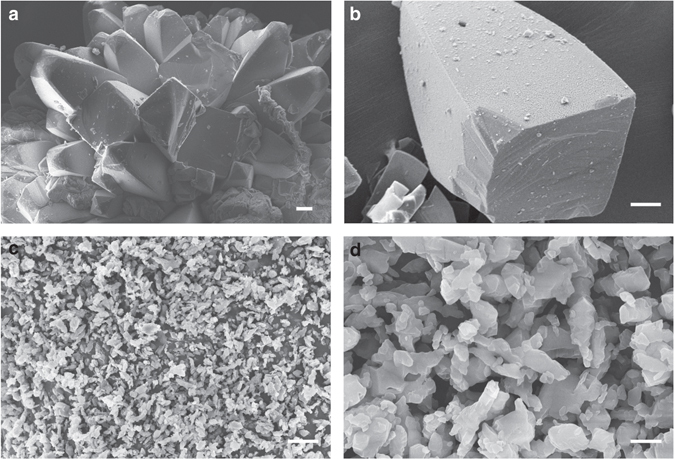
Scanning electron microscope images of ICM-101. **a**, **b** ICM-101 crystals grown from DMSO. The scale bars in **a** and **b** represent 20 μm. **c**, **d** The as-synthesized powder sample. The scale bar in **c** represents 10 μm and the scale bar in **d** represents 2 μm

To understand the morphology of ICM-101, both crystal and powdered samples were examined via scanning electron microscopy (SEM). As can be observed from the images in Fig. 3a, b, ICM-101 crystals that were grown from DMSO exhibited the prism-like morphology with smooth surface and were liable to pack into flower-shaped glomerocryst microstructures with a size of ~400 μm in the regions of severe agglomeration. In contrast with the ICM-101 crystals, the as-synthesized powdered samples displayed irregular flake morphology and the particle sizes were in the range of 2–5 μm with loose agglomeration (Fig. 3c, d). These results also indicate that crystallization can significantly change the morphology of ICM-101 (crystal shape, size, surface, etc.), and hence could provide a fresh opportunity to tune the detonation performance of ICM-101 with enhanced safety.

In general, high density of HEDMs is the result of close crystal packing. In this case, ICM-101 displayed a surprisingly high crystal density of 1.99 g cm^−3^ at 298 K (a measured density of 1.9997 g cm^−3^ determined by a gas pycnometer). The calculated packing coefficient is up to 81.0%, which is higher than that of TATB (78.1%) and FOX-7 (78.0%), suggesting a remarkably close layered stacking in the solid state^25^. We suspect that the high packing coefficient and density are attributed not only to the planar molecule structure but also to the less rotating side-groups in ICM-101. The C1-N2 and C1a-N2a bonds possess double-bond character which prevents rotation. In addition, the rotation of nitro groups is also restricted by the interactions between the protons and O2/O2a. As supported by theoretical calculations, the relative energies of ICM-101 as a function of the rotation of the nitro groups (R1) are thermally not favorable, and the corresponding values were in the range of 0–83.2 kJ mol^−1^ (Fig. 2c and Supplementary Tables 5 and 6). In contrast, if protons were bound to the N2/N2a positions, the relative energies of ICM-101 tautomerism (protons bound to N2/N2a) as a function of the rotation of nitro groups (R2) and nitroamine groups (R3) were much lower and were in the range of −11.4 to 17.0 kJ mol^−1^ for R2 and −1.9 to −61.0 kJ mol^−1^ for R3. On the basis of these calculations, it may be concluded that the rotations of the nitro groups of ICM-101 is highly restricted and aids in achieving the highly ordered packing in the solid state. In addition to the high packing coefficient, intermolecular and intramolecular bonding interactions also have a key role in attaining high density. As shown in Supplementary Figs 6 and 7, extensive strong intermolecular hydrogen bonding can be found between hydrogen and the nitro group of adjacent molecules within the layers, and the length of the hydrogen bond (HB), −NO_2_∙∙∙H, is 2.077 Å. AIM analysis reveals that the total intermolecular hydrogen bonding energy (*E*_HB_) is up to 55.6 kJ mol^−1^, which is close to the summed intremolecular *E*_HB_ of one 2,4,6-trinitrobenzene-1,3,5-triamine (TATB) molecule^26^, indicating that the intralayer interactions of ICM-101 are primarily dominated by intermolecular hydrogen bonding. It is to be noted that extremely strong *π*-type interactions exist between two adjacent layers due to the parallel near-planar structure of ICM-101. The interlayer distance in ICM-101 is about 3.20 Å, which is slightly longer than on TATB (3.1 Å) and the same as in FOX-7 (3.2 Å)^27^.

**Fig. 4:**
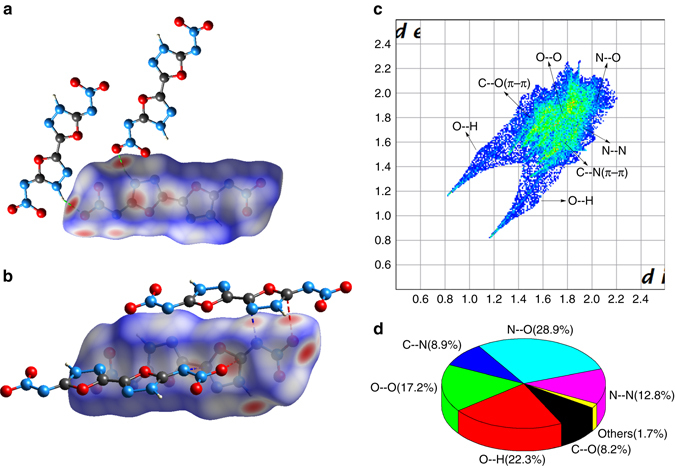
Hirshfeld surfaces calculation of ICM-101. **a** Hydrogen bond interaction of Hirshfeld surfaces of ICM-101. **b** *π*-type interaction of Hirshfeld surfaces of ICM-101. **c** Two-dimensional fingerprint plots in the crystal stacking of ICM-101. **d** The individual atomic contact percentage contribution to the Hirshfeld surface

To obtain a better understanding of ultra-high density, the two-dimensional (2D)-fingerprint of crystals and the associated Hirshfeld surfaces were employed to demonstrate the intermolecular interactions. According to the definition of Hirshfeld surfaces, the red and blue on the surfaces denotes the high and low close contact populations, respectively^28^. We could summarize two critical features from Fig. 4a, b. Firstly, the molecule shows a planar π-conjugated structure and appears in plate shapes. Secondly, most red dots located on the edges primarily denote the intermolecular HB interactions, whereas those on the plate faces usually denote π–π stacking, such as O–C and C–N interactions. These two features of Hirshfeld surfaces of ICM-101 are in accordance with the above discussions on crystal packing that the intralayer intermolecular HBs support the layers to form π–π stacking providing a plausible reason for high density of ICM-101. This can also be ascertained by the two-dimensional fingerprint plots in Fig. 4c, d. A pair of remarkable spikes on bottom left (O–H and H–O interactions constitute 22.3% of the total weak interactions) in the 2D fingerprint plots of the crystal denote HB among neighboring intralayer molecules. It is to be noted that strong C–O and C–N interactions were observed which is reflected in strong π–π stacking interaction. A high ratio of N–O interactions is observed (28.9%) which is reflected in high percentage of interlayer contact. In other words, the interlayer distance of ICM-101 is short and the packing of the crystal is exceedingly close.

### Physicochemical properties

After ICM-101 synthesis, we turned our attention to study its properties and detonation performance. The thermal stability was one of our primary concerns since it is a particularly crucial property of HEDMs. The thermal decomposition temperature (*T*_d_) of ICM-101 was determined by DSC. As shown from the DSC curve of ICM-101 (Supplementary Fig. 8), there was only one intense exothermic signal (210.4 °C, onset) with a DSC peak exotherm of 215.9 °C which corresponds to the thermal decomposition of ICM-101. The TG curve also revealed that ICM-101 starts to decompose at approximately 210 °C (a thermal explosion at 214.3 °C, Supplementary Fig. 9). These results signify that ICM-101 exhibits a rather high decomposition temperature comparable to that of RDX and CL-20 (210 °C and 215 °C, respectively, Table 1). Previous studies have demonstrated that the bond dissociation enthalpy (BDEs) for the trigger bond (i.e., the first bond to break in the molecule) is an important indicator for pyrogenic decomposition^29^. To determine the thermal stability of ICM-101, the BDE of the molecule was calculated, and the N-nitro bonds were found to be the trigger bond. The BDE of the *N*-nitro bond in ICM-101 was estimated to be around 216 kJ mol^−1^, which was in agreement with experimental observations, and is higher than that of RDX (161.0 kJ mol^−1^)^29^. As mentioned above, ICM-101 possesses a low solubility in water. With regards to the practical applications, there is a need to find an appropriate solvent for the crystal engineering of ICM-101 as it is necessary to obtain high-quality crystals of ICM-101. Hence, we estimated the solubility of ICM-101 in common organic solvents. Ten common organic solvents were tested, and the solubility results are listed in Supplementary Table 7. As compared to the solubility of 260 mg of ICM-101 in 100 g water, ICM-101 exhibited a comparatively lower solubility of <100 mg per 100 g in most organic solvents including ethyl alcohol, ethyl acetate, dichloromethane, acetonitrile, and ethers. Even in the solvent of *N*,*N*-dimethylformamide, ICM-101 was only slightly soluble at room temperature (*ca*. 1 g per 100 g) but had partial solubility in DMSO (*ca*. 9 g per 100 g). We speculate that the extensive hydrogen bonding interactions within ICM-101 play a crucial role behind low solubility in most organic solvents.

**Table 1:** Physical properties of ICM-101 and comparison with HMX, RDX and ε-CL-20

Comp.	*T*_d_^a^ *(°C)*	*d* ^b^ (*g* *cm* ^−*3*^ *)*	*Δ*_f_ *H* ^c^ *(kJ* *mol* ^−*1*^ *)*	*P* ^d^ *(GPa)*	*v*_D_^e^ *(m* *s* ^−*1*^ *)*	*IS* ^f^ *(J)*	*FS* ^g^ *(N)*	*OB* ^h^ *(%)*
ICM-101	210	1.99^i^	166.8 (159.52)	41.9 (40.39)^j^	9481 (9475)^j^	5	60	6
HMX^k^	279	1.90	116.1	41.5	9221	7	112	0
RDX^k^	210	1.81	86.3	38.0	8983	7	120	0
ε-CL-20^k^	215	2.04	365.4	46.7	9455	4	48	11

^CO, carbon monoxide; XRD, X-ray diffraction^

^a^Decomposition temperature

^b^Density based on XRD at 298 K

^c^Formation heat

^d^Detonation pressure

^e^Detonation velocity

^f^Impact sensitivity

^g^Friction sensitivity

^h^Oxygen balance based on CO

^i^The measured density by a gas pycnometer at 298 K is 1.9997 g cm^−3^

^j^The EXPLO5 calculations of detonation velocity and pressure in parenthesis are based on the measured density and the bomb calorimetry results

^k^Ref. ^13^

The heat of formation (*Δ*_f_ *H*) of ICM-101 was calculated by the isodesmic reaction approach using Gaussian 09 (Revision D.01) suite program^30^, and was estimated to be 168.8 kJ mol^−1^ (see Supplementary Fig. 10 and Supplementary Note 1 and Supplementary Table 8). In addition to the isodesmic reaction approach, Bomb calorimetry is also a well-established experimental technique to measure energies of combustion and calculated the heat of formation. Therefore, the Bomb Calorimetry study for ICM-101 was also determined by an oxygen bomb calorimetry, and the measured constant volume combustion energies was −7886 J g^−1^ (−2035.50 kJ mol^−1^). On the basis of the calculated constant volume combustion enthalpy and the Hess’s law, the standard formation enthalpy of ICM-101 was back-calculated be 159.52 kJ mol^−1^ (see Supplementary Table 9 and Supplementary Note 2), due to the unavoidable heat exchange between the calorimeter and its surroundings, this value is slightly lower than the calculated value (166.8 kJ mol^−1^). Using the measured ambient temperature density and the calculated heat of formation (or the value based on bomb calorimetry), the detonation velocity (*v*_D_) and the detonation pressure (*P*) of ICM-101 were evaluated using EXPLO5 (v6.02) program^31^. As shown in Table 1, the calculated detonation velocity and pressure of ICM-101 are 9481 m s^−1^ and 41.9 GPa, respectively, which approaches that of CL-20 (9455 m s^−1^ and 46.7 GPa, respectively) while outperforms RDX (8983 m s^−1^ and 38.0 GPa) and HMX (9221 m s^−1^ and 41.5 GPa)^13^. On the basic carbon monoxide (CO), ICM-101 exhibits a positive *OB* value of 6%, which is higher than that of RDX (0%) or HMX (0%) and close to that of CL-20 (11%). The detonation properties coupled with rather good thermal stability and low water solubility make ICM-101 an attractive candidate as high performing HEDMs.

**Fig. 5:**
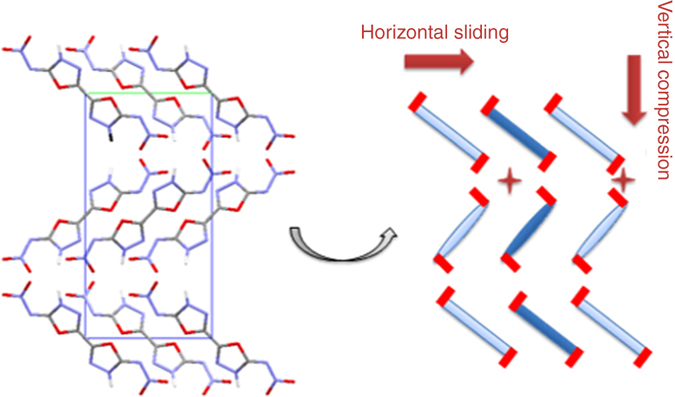
Packing diagram of ICM-101. Viewed down the crystallograph from a-axis

In addition to detonation properties, the impact and friction sensitivities of ICM-101 were evaluated using a standard BAM fall-hammer and a BAM friction tester, respectively. Due to a few promising structural features including extensive intermolecular HBs and layered stacking in the crystal, ICM-101 was expected to have a low sensitivity towards mechanical stimuli. However, unexpectedly, high sensitivities of ICM-101 towards mechanical stimuli were found, i.e., the impact sensitivity (IS) and friction sensitivity (FS) were estimated to be 5 J and of 60 N, respectively. These values are higher than those of HMX (7 J and 112 N), but lower than those of CL-20 (4 J and 48 N). The interlayer sliding within a crystal lattice is a feature that contributes to the insensitivity of TATB, FOX-7, and 2,6-diamino-3,5-dinitropyrazine 1-oxide (LLM-105), which have graphite-like stacking that allows such sliding. However, the crossing of interlayer molecules in the a-axis direction may be a factor that prevents ICM-101 from the better sliding as the molecules such as LLM-105 or TATB (Fig. 5). The calculated deformation potential of ICM-101 is provided in Supplementary Fig. 11. It was found that the horizontal sliding causes a low deformation potential, while the vertical compression energy variation of ICM-101 is relatively high.

## Discussion

We demonstrate the synthesis and characterization of an energetic HEDM ICM-101. This compound is determined via single-crystal XRD and shows a planar molecular structure, high packing coefficient, remarkable hydrogen bonding interaction, and interlayer π–π stacking interaction which contribute to a particularly high density. The compound ICM-101 presents itself as a fresh example of CHNO-based HEDMs with a crystal density as high as 2.037 g cm^−3^ at 170 K. This neutral compound possesses decent thermal stability (210.4 °C as onset decomposition temperature), poor solubility in water and most organic solvents. In addition, this prepared energetic material is both nitrogen- and oxygen-rich, as shown by the values of their detonation performances (*v*_D_ = 9481 m s^−1^; *P* = 41.9 GPa) calculated by using the EXPLO5 6.01 program. The combination of good physical properties and detonation performance as well as its straightforward preparation highlight its great promise of potential applications as HEDM.

## Methods

### General

Caution, the compound ICM-101 is a highly energetic material and tends to explode under physical stress. Laboratories and personnel must be properly grounded, and safety equipment such as protective gloves and coats, face shield and explosion-proof baffle are recommended.

### Materials

Cyanogen bromide, (98%) and oxalyl dihydrazide, (97%) were purchased from *J*&*K*. Other commercial reagents were used as received.

### Product characterization

^1^H and ^13^C NMR spectra were measured at 600 MHz (Bruker AVANCE 600) with DMSO-d_6_ as the solvent. IR spectra were recorded on a PerkinElmer Spectrum Two IR Spectrometer. High resolution mass spectra were performed on a Shimadzu LCMS-IT-TOF mass spectrometer using electrospray ionization (ESI). Elemental analysis was performed on a Vario Micro cube elemental analyzer. Thermal property measurements were carried out on a TGA/DSC Mettler Toledo calorimeter equipped with an auto cool accessory. Impact and friction sensitivity measurements were made using a standard BAM Fall hammer and a BAM friction tester. The constant-volume combustion energy of ICM-101 was determined by an oxygen bomb calorimetry (IKA C5000). The morphology of ICM-101 was examined by field emission scanning electron microscopy (FE-SEM, Ultra-55, Carl Zeiss, Germany). X-ray powder diffraction (PXRD) analysis was performed on a Bruker D8 Advance X-ray powder diffractometer. The heat of formation and detonation properties were calculated with the Gaussian 09 and Explo5 (version 6.02) software, respectively.

### Bis(nitroamino-1,3,4-oxadiazole)

Synthetic methodology of various derivatives of 5,5′-dihydrazinyl-2,2′-bi(1,3,4-oxadiazole) has been reported through a condensation reaction between oxalyl dihydrazide and carbon disulphide in alkaline medium^32^. In this work, we developed a new construction strategy for the synthesis of [2,2′-bi(1,3,4-oxadiazole)]-5,5′-diamine. Oxalyl dihydrazide (1.18 g, 10 mmol) was dissolved in methanol (100 ml) and potassium bicarbonate (2.21 g, 22 mmol) was added in one portion at 0 °C. Cyanogen bromide (2.12 g, 20 mmol) was added to the mixture. The suspension was stirred at 0 °C for 4 h, heated up to 20 °C and stirred for 16 h. And then water (250 ml) was added. After that, the mixture was stirred for another 3 h. The precipitate was collected and washed with ice water. The precipitate was dried to afford 1.52 g of a light yellow solid (compound **1**, 90.5% yield). This solid was used directly in the next step without further purification. Compound **1** (1 g, 5.95 mmol) was added by portions into smoothly stirred fuming HNO_3_ (8 ml) which was cooled by ice. Then, the reaction was allowed to warm to room temperature and continued for about 24 h. After a white precipitate appeared, the mixture was poured into ice water and filtered to obtain a white precipitate. After washing with small amount of water, the white solid was dried naturally, and pure ICM-101 was obtained as a white powder (66.8% yield); *T*_dec_: 210 °C ^1^H NMR (600 MHz, DMSO-d_6_): *δ* ppm: 13.74 (brs, 2H, NH); ^13^C NMR (151 MHz, DMSO-d_6_): *δ* ppm: 163.64, 145.93 (the images of ^1^H and ^13^C NMR spectra are provided in Supplementary Figs 12 and 13); IR (KBr): *γ* 1588, 1490, 1302, 1246, 1157, 1075, 964, 661; ESI-HRMS: *m/z* anion calcd for C_4_HN_8_O_6_ [M]^−^: 257.0025, found: 257.0027; elemental analysis calcd (%) for C_4_H_2_N_8_O_6_ (258.01): C 18.61, H 0.78, N 43.41, found: C 18.63, H 0.80, N 43.78.

### Data availability

The data that support the findings of this study are available from the corresponding authors on request.

## Electronic supplementary material

Supplementary Information

## References

[1] Giles J (2004). Green explosives: collateral damage. Nature..

[2] Huynh MHV, Coburn MD, Meyer TJ, Wetzler M (2006). Green primary explosives: 5-Nitrotetrazolato-N2-ferrate hierarchies. Proc. Natl Acad. Sci.USA.

[3] Huynh MHV, Hiskey MA, Meyer TJ, Wetzler M (2006). Green primaries: Environmentally friendly energetic complexes. Proc. Natl Acad. Sci. USA.

[4] Thottempudi V, Shreeve JM (2011). Synthesis and promising properties of a new family of high-fensity rnergetic salts of 5-nitro-3-trinitromethyl-1H-1,2,4-triazole and5,5′-bis(trinitromethyl)-3,3′-azo-1H-1,2,4-triazole. J. Am. Chem. Soc..

[5] Göbel M, Tchitchanov BH, Murray JS, Politzer P, Klapötke TM (2009). Chlorotrinitromethane and its exceptionally short carbon–chlorine bond. Nature Chem.

[6] Zhang C, Sun C, Hu B, Yu C, Lu M (2016). Synthesis and characterization of the pentazolate anion cyclo-N_5_^−^in (N_5_)_6_(H_3_O)_3_(NH_4_)_4_Cl. Science.

[7] Bélanger-Chabot G, Rahm M, Haiges R, Christe KO (2015). Ammonia–(dinitramido)boranes: high-energy-density materials. Angew. Chem. Int. Ed. Rngl..

[8] Fischer D, Klapötke TM, Stierstorfer J (2015). 1,5-Di(nitramino)tetrazole: high sensitivity and superior explosive performance. Angew. Chem Int. Ed. Engl...

[9] Zhang J, Shreeve JM (2014). 3,3′-Dinitroamino-4,4′-azoxyfurazan and its derivatives: an assembly of diverse N-O building blocks for high-performance energetic materials. J. Am. Chem. Soc..

[10] Zhang M, Eaton PE, Gilardi R (2000). Hepta- and octanitrocubanes. Angew. Chem. Int. Ed. Engl..

[11] Zhao XX (2016). Design and synthesis of energetic materials towards high density and positive oxygen balance by N-dinitromethyl functionalization of nitroazoles. J. Mater. Chem. A.

[12] Klapötke TM, Mayr N, Stierstorfer J, Weyrauther M (2014). Maximum compaction of ionic organic explosives: bis(hydroxylammonium) 5,5′-dinitromethyl-3,3′-bis(1,2,4-oxadiazolate) and its derivatives. Chem. Eur. J..

[13] Fischer N, Fischer D, Klapötke TM, Piercey DG, Stierstorfer J (2012). Pushing the limits of energetic materials - the synthesis and characterization of dihydroxylammonium 5,5′-bistetrazole-1,1′-diolate. J. Mater. Chem..

[14] Bennion JC, Chowdhury N, Kampf JW, Matzger AJ (2016). Hydrogen peroxide solvates of 2,4,6,8,10,12-hexanitro-2,4,6,8,10,12-hexaazaisowurtzitane. Angew. Chem. Int. Ed. Engl..

[15] Cui K (2014). Synthesis and characterization of a thermally and hydrolytically stable energetic material based on N-Nitrourea. Propell. Explos. Pyrot..

[16] Klenov MS (2016). Synthesis of tetrazino-tetrazine 1,3,6,8-tetraoxide (TTTO). Angew Chem. Int. Ed. Engl..

[17] Politzer P, Lane P, Murray JS (2013). Computational characterization of two di-1,2,3,4-tetrazine tetraoxides, DTTO and iso-DTTO, as potential energetic compounds. Cent. Eur. J. Energ. Mater..

[18] Christe KO (2015). Are DTTO and iso-DTTO worthwhile targets for synthesis?. Propell. Explos. Pyrot.

[19] Mendoza-Cortes JL, An Q, Goddard WA, Ye C, Zybin S (2016). Prediction of the crystal packing of di-tetrazine-tetroxide (DTTO) energetic material. J. Comput. Chem..

[20] Bapat VK, Skider AK, Mehilal, Polke BG, Agrawal JP (2000). Synthesis and characterization of 2,4,6-tris(3′,5′-diamino-2′,4′,6′-trinitrophenylamino)-1,3,5- triazine (PL-1): a new thermally stable insensitive high explosive. J. Energ. Mater..

[21] Fischer D, Klapötke TM, Stierstorfer J (2014). Potassium 1,1′-dinitramino-5,5′-bistetrazolate: a primary explosive with fast detonation and high initiation power. Angew Chem. Int. Ed. Engl..

[22] Tang Y, Zhang J, Mitchell LA, Parrish DA, Shreeve JM (2015). Taming of 3,4-di(nitramino)furazan. J. Am. Chem. Soc..

[23] Sikder AK, Sikder N (2004). A review of advanced high performance, insensitive and thermally stable energetic materials emerging for military and space applications. J. Hazard. Mater..

[24] Zhang J, Dharavath S, Mitchell LA, Parrish DA, Shreeve JM (2016). Energetic salts based on 3,5-bis(dinitromethyl)-1,2,4-triazole monoanion and dianion: controllable preparation, characterization, and high performance. J. Am. Chem. Soc..

[25] Zhang J, Zhang Q, Vo TT, Parrish DA, Shreeve JM (2015). Energetic salts with π-stacking and hydrogen-bonding interactions lead the way to future energetic materials. J. Am. Chem. Soc..

[26] Ma Y (2014). Crystal packing of low-sensitivity and high-energy explosives. Cryst. Growth Des..

[27] Zhang J, Mitchell LA, Parrish DA, Shreeve JM (2015). Enforced layer-by-layer stacking of energetic salts towards high-performance insensitive energetic materials. J. Am. Chem. Soc..

[28] Spackman MA, Jayatilake D (2009). Hirshfeld surface analysis. CrystEngComm..

[29] Zhang J, Parrish DA, Shreeve JM (2014). Thermally stable 3,6-dinitropyrazolo [4,3-c]pyrazole-based energetic materials. Chem. Asian J..

[30] Frisch, M. J. et al. *Gaussian 09, Revision D. 01* (Gaussian Inc., 2009).

[31] Sućeska, M. *EXPLO5 6.02* (Brodarski Institure, 2009).

[32] Jani MK, Undavia NK, Trivedi PB (1989). Synthesis of Bis(1,3,4-oxadiazol-2-yl)-5-aryl-hydrazones. J. Indian Chem. Soc..

