# Supplementary material for: A promising high-energy-density material
Source: Nat Commun. 2017 Aug 3;8:181. doi: 10.1038/s41467-017-00286-0 (PMC5541047; doi:10.1038/s41467-017-00286-0)
Supplement: Supplementary file 1 — Supplementary Information [file 41467_2017_286_MOESM1_ESM.pdf]

File Name: Supplementary Information
Description: Supplementary Figures, Supplementary Tables, Supplementary Notes and Supplementary References

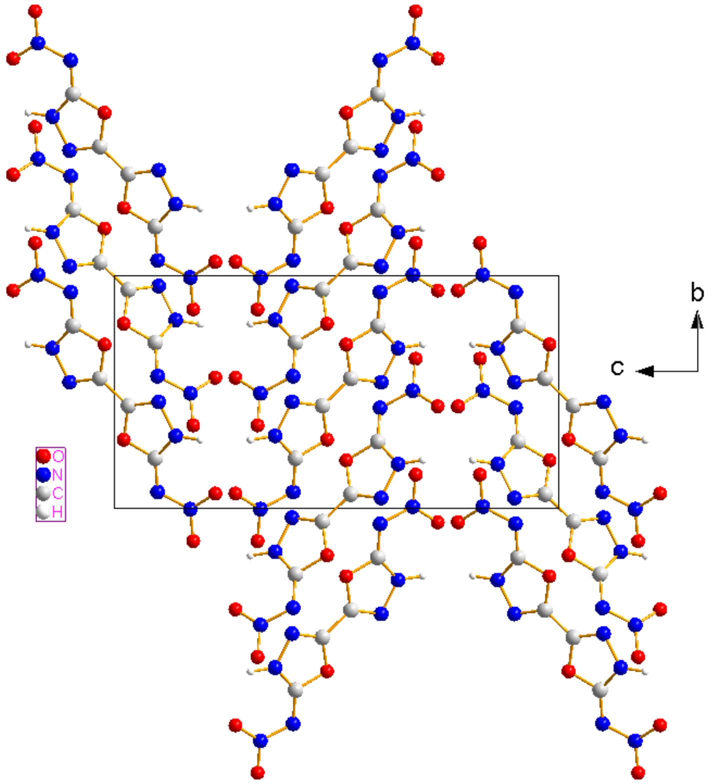

Supplementary Figure 1. **X-ray crystal structure of ICM-101.** Single molecular view along the **a** axis.

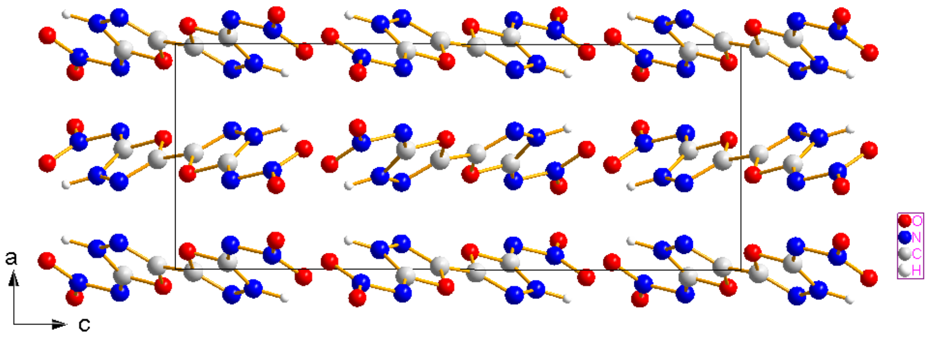

Supplementary Figure 2. **X-ray crystal structure of ICM-101.** Single molecular view along the **b** axis.

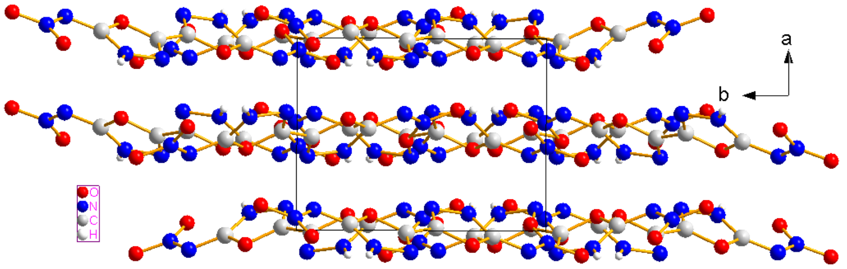

Supplementary Figure 3. X-ray crystal structure of ICM-101. Single molecular view along the **c** axis.

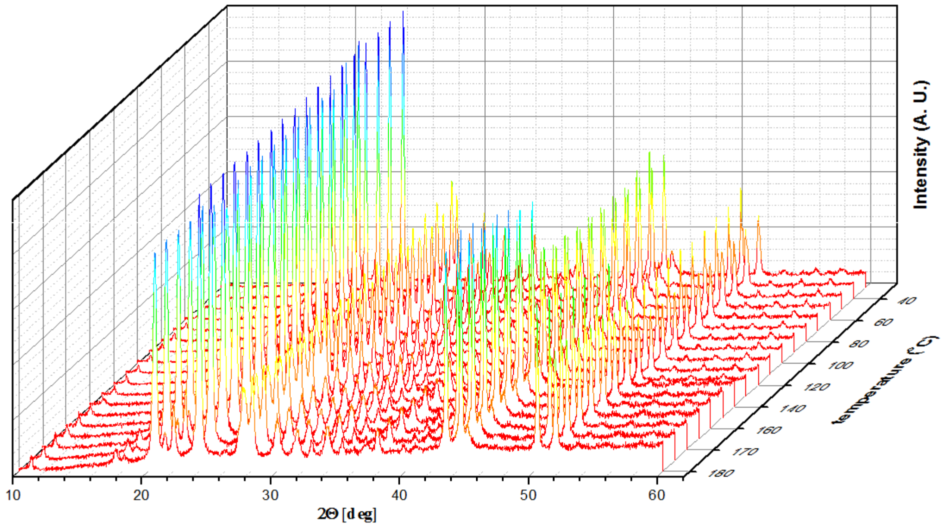

Supplementary Figure 4. Powder X-ray diffraction patterns of ICM-101. The possible polymorph transitions of ICM-101 were investigated using temperature-dependent XRD technology over the temperature range of 30 °C to 180 °C.

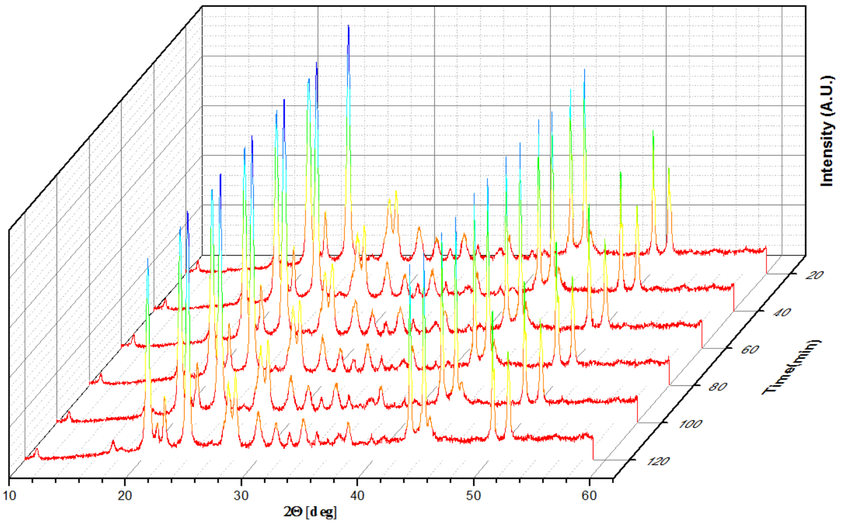

Supplementary Figure 5. Powder X-ray diffraction patterns of ICM-101. The possible polymorph transitions of ICM-101 were investigated using temperature-dependent XRD technology upon heating at 170°C for 2 hours.

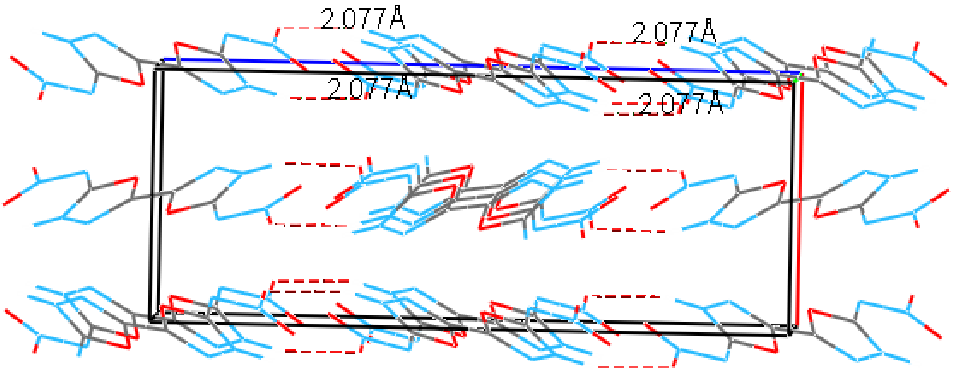

Supplementary Figure 6. Intralayered hydrogen bonding interactions.

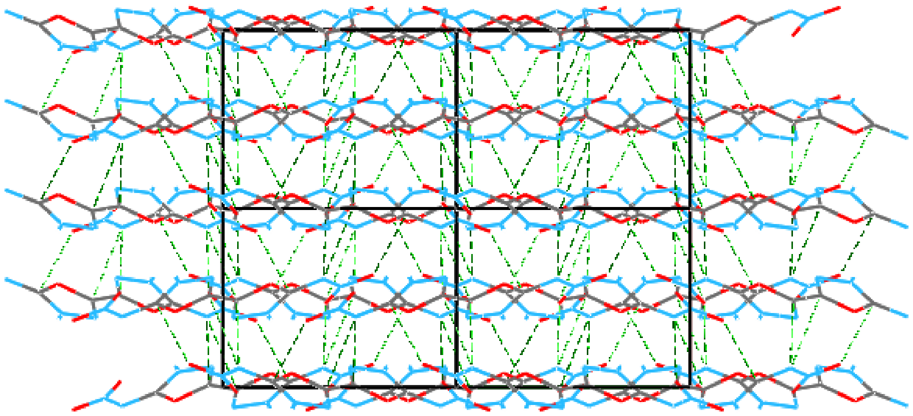

Supplementary Figure 7. Interlayered hydrogen bonding interactions.

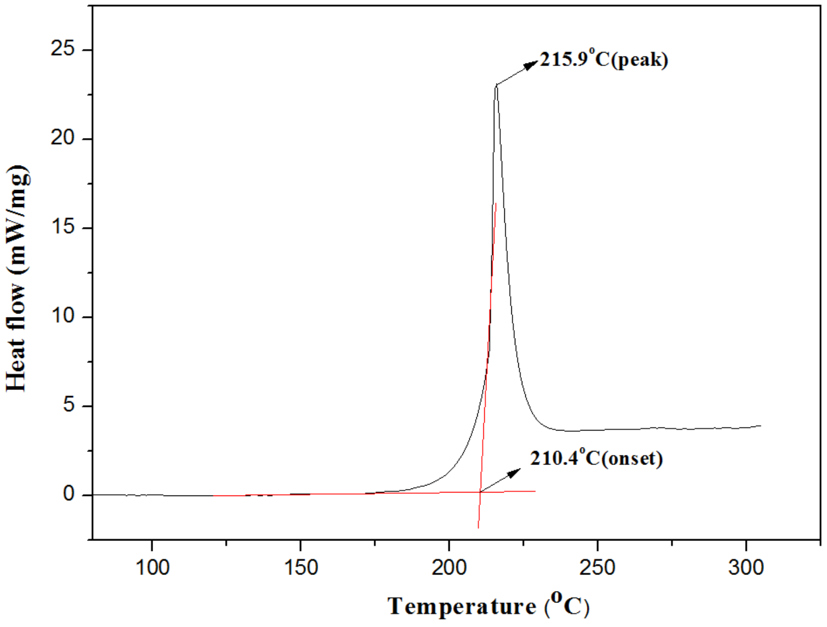

Supplementary Figure 8. DSC curve of ICM-101. The DSC plots were recorded at the heating rate of 10 °C min^{-1}.

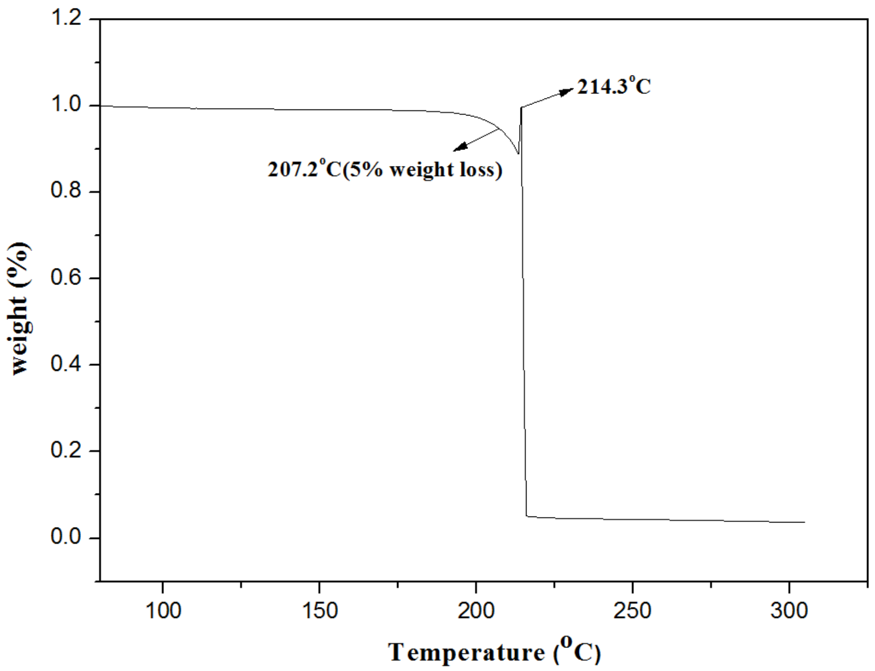

Supplementary Figure 9. TGA curve of ICM-101. The TGA plots were recorded at the heating rate of 10°C min^{-1}.

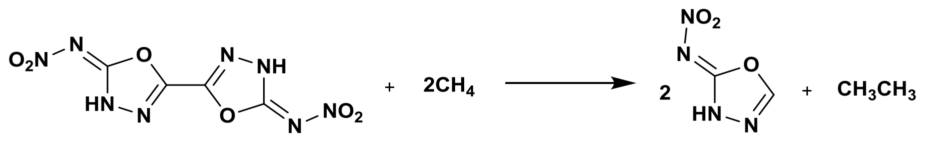

Supplementary Figure 10. Isodesmic reaction of ICM-101.

<u>Supplementary Note 1. The theoretic calculation about heat of formation of ICM-101</u>.

Theoretical calculations were performed by using the Gaussian 09 (Revision D.01) suite of programs.[1] The geometric optimization and frequency analyses were completed by using the B3LYP functional with the 6-31+G** basis set. Single energy points were calculated at the MP2/6-311++G** level of theory. For all of the compounds, the optimized structures were characterized to be true local energy minima on the potential-energy surface without imaginary frequencies. The isodesmic reaction was carried out to obtain the gas-phase heat of formation of the neutral compound. The gas-phase enthalpies of the building-block molecules were obtained by using the atomization method with the G2 ab initio calculations. Then the remaining task is to determine the solid-state heat of formation for the synthesized compound.

The solid-state enthalpy of formation for neutral compound can be estimated by subtracting the heat of sublimation from gas-phase heat of formation. On the basis of the literature,[2,3] the heat of sublimation can be estimated with Trouton's rule according to supplementary equation 1, where T represents either the melting point or the decomposition temperature when no melting occurs prior to decomposition:

$$\Delta H_{sub} = 188/\text{J mol}^{-1}\text{K}^{-1} \times T \qquad 1$$

In this work, the crystal calculation was done by using Crystal Explorer software.[4] The deformation potential can be expressed by energy induced pressure and the energy was obtained from the single point energy difference before and after deformation. For convenient comparison, the value is converted from mol units into volume units by dividing by the unit cell volume supplementary equation 2.

$$P = (E_{\text{after def}} - E_{\text{before def}})/V_{\text{unit cell}} \qquad 2$$

In this part of the calculations, single point energies were obtained from optimized structures using BLYP-D3/def2-QZVPP method [5] using ORCA 3.0.[6]

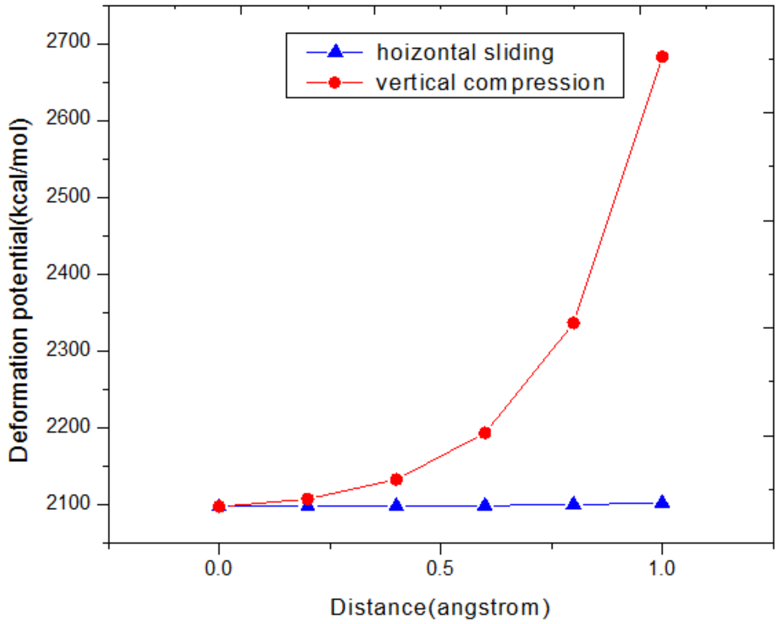

Supplementary Figure 11. Internal stress curve (deformation potential). Along the horizontal sliding and vertical compression.

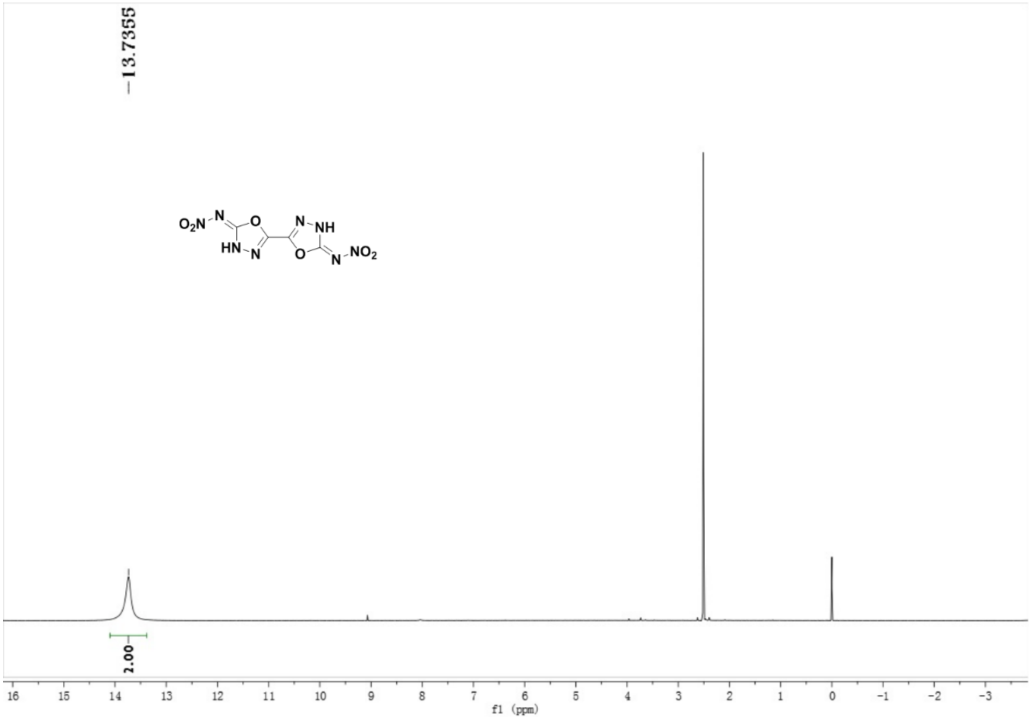

Supplementary Figure 12. ^{1}**H NMR spectrum of ICM-101.**

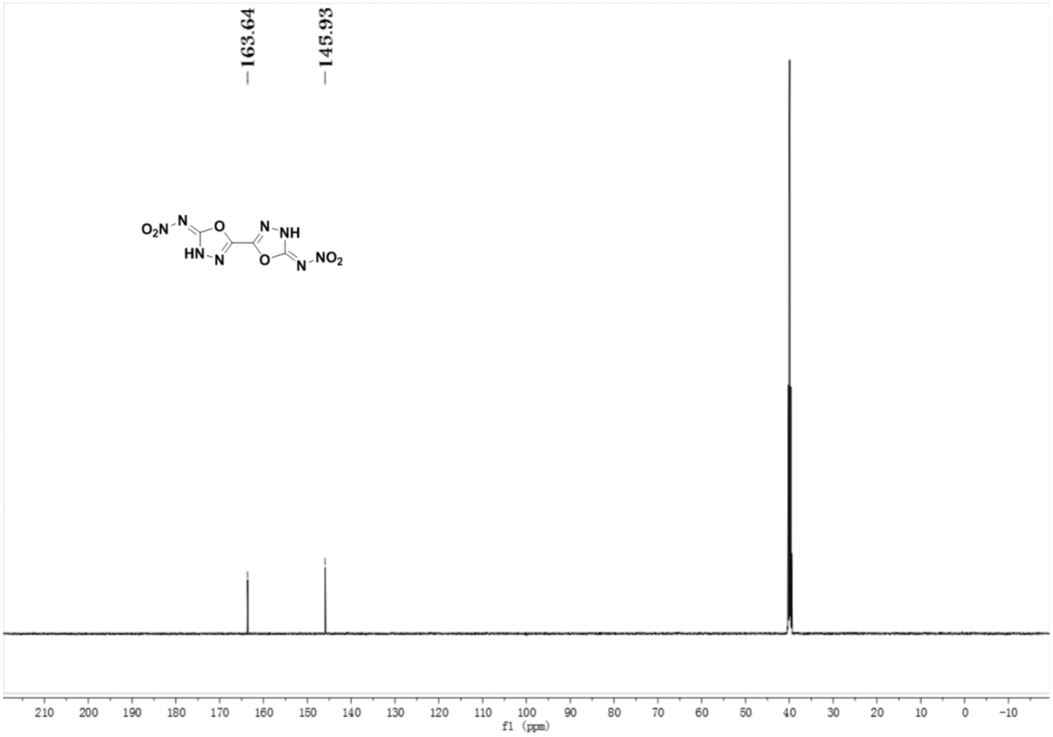

Supplementary Figure 13. ^{13}**C NMR spectrum of ICM-101.**

Supplementary Table 1. **Crystallographic data for energetic molecule ICM-101.** Single crystal X-ray diffraction data was collected on an Oxford Xcalibur diffratometer with Mo KR monochromated radiation (λ=0.71073 Å). The crystal structures were solved by direct methods. The structures were refined on F2 by full-matrix least-squares methods using the SHELXTL program package.[7] All non-hydrogen atoms were refined anisotropoically.

CCDC	1523415
Formula	$C_4H_2N_8O_6$
Mr	258.14
crystal system	orthorhombic
space group	*Pbca*
a [Å]	6.4008(14)
b [Å]	8.3648(17)
c [Å]	16.094(3)
α [Å]	90
β [Å]	90
γ [Å]	90
V [Å^3]	861.7(3)
Z	4
T [K]	298
ρ[g·cm^{-3}]	1.990
Mu [mm^{-1}]	0.185
F(000)	520.0
θ [°]	2.531 to 27.555
index range	$-8 \leq h \leq 8$
	$-8 \leq k \leq 10$
	$-20 \leq l \leq 16$
reflections collected	4924
independent reflections	993 [R_{int} = 0.1035, R_{sigma} = 0.0669]
data/restraints/parameters	993/0/86
GOF on F^2	1.001
R_1 [I>2σ(I)]	0.0452
wR_2 [I>2σ(I)]	0.0981
R_1(all data)	0.0818
wR_2(all data)	0.1098
largest diff. peak and hole [e Å^{-3}]	0.51/-0.41

Supplementary Table 2. Bond lengths for ICM-101.

Bond	Length/Å
O(3)-C(1)	1.359(2)
O(3)-C(2)	1.364(2)
O(1)-N(1)	1.231(2)
O(2)-N(1)	1.227(2)
N(4)-N(3)	1.381(3)
N(4)-C(2)	1.277(3)
N(3)-C(1)	1.331(3)
N(1)-N(2)	1.364(2)
N(2)-C(1)	1.311(3)
C(2)-C(2)’	1.436(4)

Supplementary Table 3. Bond angles for ICM-101.

Bond	Angle/º
C(1)-O(3)-C(2)	104.41(15)
C(2)-N(4)-N(3)	103.02(17)
C(1)-N(3)-N(4)	111.27(18)
O(1)-N(1)-N(2)	115.53(17)
O(2)-N(1)-O(1)	122.56(17)
O(2)-N(1)-N(2)	121.91(19)
C(1)-N(2)-N(1)	113.91(17)
N(3)-C(1)-O(3)	106.68(19)
N(2)-C(1)-O(3)	115.89(17)
N(2)-C(1)-N(3)	137.42(19)
O(3)-C(2)-C(2)'	118.4(2)
N(4)-C(2)-O(3)	114.58(17)
N(4)-C(2)-C(2)'	127.0(2)

Supplementary Table 4. Torsion angles for ICM-101.

Bond	Torsion angle/º
O(1)-N(1)-N(2)-C(1)	178.71(17)
O(2)-N(1)-N(2)-C(1)	-2.0(3)
N(4)-N(3)-C(1)-O(3)	-2.1(2)
N(4)-N(3)-C(1)-N(2)	178.8(2)
N(3)-N(4)-C(2)-O(3)	0.2(2)
N(3)-N(4)-C(2)-C(2)’	-179.2(3)
N(1)-N(2)-C(1)-O(3)	177.38(16)
N(1)-N(2)-C(1)-N(3)	-3.6(3)
C(1)-O(3)-C(2)-N(4)	-1.4(2)
C(1)-O(3)-C(2)-C(2)’	178.0(2)
C(2)-O(3)-C(1)-N(3)	2.0(2)
C(2)-O(3)-C(1)-N(2)	-178.66(17)
C(2)-N(4)-N(3)-C(1)	1.2(2)

Supplementary Table 5. The geometry and XYZ coordinates of ICM-101. Symbolic Z-matrix: Charge = 0 Multiplicity = 1.

Atom	X	Y	Z
C	-4.55311	-1.14569	-0.90768
C	-5.63954	-0.54061	0.86242
O	-4.37779	-1.1344	0.50386
N	-5.51682	-0.12951	-1.30986
H	-5.06188	0.72378	-1.56466
N	-6.26888	0.01422	-0.13859
C	-6.20453	-0.5717	2.2947
C	-7.63874	-0.6244	3.91334
O	-7.47362	0.0082	2.65007
B	-6.34646	-1.01914	4.45861
H	-5.97998	-0.31879	5.07116
N	-5.58185	-1.13913	3.29281
N	-8.77752	-0.83281	4.49052
N	-3.95201	-1.95426	-1.71904
N	-4.20571	-1.88305	-3.09401
O	-5.00507	-1.04655	-3.53326
O	-3.63063	-2.65662	-3.87025
N	-8.82499	-1.47365	5.73433
O	-9.91447	-1.67304	6.28653
O	-7.77746	-1.84078	6.28166

Supplementary Table 6. The geometry and XYZ coordinates of ICM-101 tautomer. Protons bound to N2/N2a, the initial structure was set up as planar (rotation angle equal zero). Symbolic Z-matrix: Charge = 0 Multiplicity = 1.

Atom	X	Y	Z
C	2.71597400	0.71079800	-0.25818500
C	0.62953800	0.50314100	-0.39712000
O	1.77259400	-0.21705600	-0.54656600
N	2.22191900	1.88097400	0.02196800
N	0.84734300	1.74717100	-0.08173100
C	-0.64243700	-0.15903900	-0.59120400
C	-2.73789400	-0.39922100	-0.66207100
O	-1.78035500	0.53795000	-0.37414600
N	-2.23051800	-1.53744400	-1.02225200
N	-0.85740300	-1.38558300	-0.98004900
N	-4.07616100	-0.06605800	-0.56053100
N	4.05265000	0.38907400	-0.40480000
N	4.39870646	-0.92588072	-0.86065425
O	3.47835685	-1.68626885	-1.09286031
O	5.59587969	-1.10688422	-1.00233954
N	-4.40677615	1.25394094	-0.15580786
O	-3.47452506	2.00401384	0.07471613
O	-5.60318226	1.47185052	-0.05232462
H	4.65482786	0.64742092	0.37153743
H	-4.75873900	-0.42260249	-1.22129396

Supplementary Table 7. The solubility of ICM-101 in common solvents. For the purpose of evaluating the solubility in common organic solvents, ten common organic solvents were tested.

**solvent**	**H_2O**	**DMSO**	**DMF**	**Acetone**	**CH_2Cl_2**	**CH_3OH**
Solubility of ICM-101 (g/100g solvent)	0.26	9	1	0.11	0.05	0.9
**solvent**	**EtOAc**	**EtOH**	**CH_3CN**	**Hexane**	**Et_2O**	**--**
Solubility of ICM-101 (g/100g solvent)	0.01	0.025	0.02	<0.01	<0.01	--

Supplementary Table 8. The calculated enthalpies of ICM-101.

**Compd.**	**ρ(g cm^{-3})**	**T_d**	**HOF_{gas}**	**HOF_{sub}**	**HOF_{solid}**
**ICM-101**	1.99	210	257.60	90.80	166.79

Supplementary Table 9. Bomb Calorimetry for ICM-101.

batch	1	2	3	4	5	6
$\Delta_c U$(J/g)	-7913	-7877	-7883	-7889	-7880	-7875

Supplementary Note 2. Calculation about heat of formation of ICM-101 by experiment.

The constant-volume combustion energy of ICM-101 was determined by an oxygen bomb calorimetry (IKA C5000). Approximately 200 mg ICM-101 was pressed with a well-define amount of benzoic acid (ca. 800 mg) to form a tablet to ensure better combustion. The recorded data are the average of six single measurements. The calorimeter was calibrated by the combustion of certified benzoic acid in an oxygen atmosphere at a pressure of 30.5 bar. The experimental data of constant-volume combustion energy (six single measurements).

$$\Delta_c \bar{U} = -7886 \text{ J g}^{-1} = -2035.50 \text{ kJ mol}^{-1} \quad 3$$

The averaged experimental value for the constant volume combustion energies ($\Delta_c \bar{U}$) of ICM-101 is -7886 J g^{-1} (-2035.50 kJ mol^{-1}). The combustion reaction equation supplementary equation 4 and energy of combustion supplementary equation 5 are listed as follows.

The combustion reaction equation supplementary equation 4:

$$C_4H_2N_8O_6\,(s) + 3/2O_2\,(g) \rightarrow 4CO_2\,(g) + H_2O\,(l) + 4N_2\,(g) \quad 4$$

The energy of combustion equation supplementary equation 5:

$$\Delta_c H^o_m\,(C_4H_2N_8O_6, s) = \Delta_c U_m + \Delta nRT \quad 5$$

where Δn is the change in the number of gas products during the reaction process, R is 8.314 J mol^{-1} K^{-1}, and T is 298.15 K. According to supplementary equation 4 and supplementary equation 5, the calculated combustion enthalpy of ICM-101 was derived to be -2019.39 kJ mol^{-1}. Based on the calculated combustion enthalpy of ICM-101 and the known enthalpies of formation of the combustion products (CO_2 (g) = -393.51 kJ mol^{-1}), H_2O (l) = -285.83 kJ mol^{-1}, and N_2 is zero), the standard formation enthalpy of ICM-101 was back-calculated from its combustion equation supplementary equation 4. On the basis of Hess's law in thermochemical supplementary equation 6, the standard enthalpy of formation ($\Delta_f H^o_m$) of ICM-101 is calculated to be 159.52 kJ mol^{-1}.

$$\Delta_f H^o_m(C_4H_2N_8O_6, s) = 4\Delta_f H^o_m(CO_2,g) + \Delta_f H^o_m(H_2O, l) - \Delta_c H^o_m(C_4H_2N_8O_6, s) \quad 6$$

Supplementary References

1. Frisch, M. J. et al., Gaussian 09, Revision D. 01, Gaussian Inc., Wallingford C T, **2009**.

2. Trouton, F. IV. On molecular latent heat. *Philos. Mag.* **18**, 54-57 (1884).

3. Westwell, M. S., Searle, M. S., Wales, D. J., Wiliams, D. H. Empirical Correlations between Thermodynamic Properties and Intermolecular Forces. *J. Am. Chem. Soc.* **117**, 5013-5015 (1995).

4. Wolff, S. K., Grimwood, D. J., McKinnon, J. J., Turner, M. J., Jayatilaka, D., Spackman, M. A. CrystalExplorer, version 3.1; University of Western Australia: Crawley, Australia, **2012**.

5. Grimme, S., Ehrlich, S., Goerigk, L. Effect of the damping function in dispersion corrected density functional theory. *J. Comput. Chem.* **32**, 1456-1465 (2011).

6 Neese, F. The ORCA program system. *WIREs Comput. Mol. Sci.* **2**, 73-78 (2012).

7 Cox, J. D., Wagman, D. D., Medvedev, V. A. CODATA Key Values for Thermodynamics, Hemisphere Publishing Corp, New York, **1989**.
